# Applications of Mesenchymal Stem Cells and Neural Crest Cells in Craniofacial Skeletal Research

**DOI:** 10.1155/2016/2849879

**Published:** 2016-02-24

**Authors:** Satoru Morikawa, Takehito Ouchi, Shinsuke Shibata, Takumi Fujimura, Hiromasa Kawana, Hideyuki Okano, Taneaki Nakagawa

**Affiliations:** ^1^Department of Dentistry and Oral Surgery, Keio University School of Medicine, 35 Shinanomachi, Shinjuku-ku, Tokyo 160-8582, Japan; ^2^Department of Physiology, Keio University School of Medicine, 35 Shinanomachi, Shinjuku-ku, Tokyo 160-8582, Japan

## Abstract

Craniofacial skeletal tissues are composed of tooth and bone, together with nerves and blood vessels. This composite material is mainly derived from neural crest cells (NCCs). The neural crest is transient embryonic tissue present during neural tube formation whose cells have high potential for migration and differentiation. Thus, NCCs are promising candidates for craniofacial tissue regeneration; however, the clinical application of NCCs is hindered by their limited accessibility. In contrast, mesenchymal stem cells (MSCs) are easily accessible in adults, have similar potential for self-renewal, and can differentiate into skeletal tissues, including bones and cartilage. Therefore, MSCs may represent good sources of stem cells for clinical use. MSCs are classically identified under adherent culture conditions, leading to contamination with other cell lineages. Previous studies have identified mouse- and human-specific MSC subsets using cell surface markers. Additionally, some studies have shown that a subset of MSCs is closely related to neural crest derivatives and endothelial cells. These MSCs may be promising candidates for regeneration of craniofacial tissues from the perspective of developmental fate. Here, we review the fundamental biology of MSCs in craniofacial research.

## 1. Introduction

Developmental origins are beginning to be elucidated through rigorous studies in stem cell biology. Recent studies have demonstrated that the basis of regenerative medicine can be found in developmental biology. Indeed, many applications in regenerative medicine mimic the development and healing of specific tissues.

Mesenchymal stem cells (MSCs) are commonly used in both basic and clinical studies because they can be easily identified in adult tissues. MSCs were first identified as fibroblast-like cells in the bone marrow [1], resemble colony forming unit-fibroblasts (CFU-Fs) at clonal density, and have the capacity for differentiation into mesenchymal lineages, such as bone, cartilage, and fat [2]. Notably, MSCs and neural crest cells (NCCs) are both used in various approaches in craniofacial biology because of their developmental similarities. Indeed, the craniofacial mesenchyme developmentally originates from NCCs [3–5], and NCCs are developmentally identified at the embryonic stages [6]. It is difficult to isolate NCCs because of their limited accessibility and ethical concerns; therefore, it is difficult to directly use NCCs in patients. In contrast, MSCs are present in easily accessible adult tissues, such as bone marrow, fat tissues, and synovium, enabling facile isolation. Importantly, MSCs and NCCs have similar self-renewal and differentiation potential, and MSCs are capable of differentiating into neuronal cells [7, 8]. Furthermore, MSCs can also differentiate into endothelial cells [9–11] and are indispensable for tissue formation [12, 13]. Similar findings have also been reported in skeletal tissues [14, 15], suggesting that adult MSCs may be useful in clinical applications associated with the regeneration of skeletal tissues, particularly because of the necessity for synchronized neural tissue formation and vascularization.

Skeletal stem cells (SSCs), which were recently identified [16, 17], have been shown to contribute to the construction of skeletal tissues during development and wound healing. However, the formation and regeneration of skeletal tissues involve not only construction of bone, but also vascularization and neural synchronization [18–20]. Despite this fact, few studies have evaluated these processes with regard to SSCs.

NCCs, MSCs, and SSCs are all isolated using culture and exhibit overlapping self-renewal and multipotent differentiation potential. Thus, clarifying the specific characteristics of each cell type will improve the clinical application of these types of stem cells. In this review, we discuss the fundamental biology of stem cells in craniofacial research.

## 2. Stem Cells in Craniofacial Research

Skeletal tissues are composed of a network of hard tissues, including bone and cartilage. The jawbone and teeth comprise the craniofacial region, and many individuals suffer from skeletal diseases, such as metastasis of oral malignant tumors into the bone, congenital craniofacial malformation, severe periodontitis, and medication-related osteonecrosis of the jaw [21]. These diseases cause eating difficulty, aesthetic disorders, respiratory distress, and speech disorders, leading to decreased quality of life. Current fundamental approaches to these diseases include surgery and subsequent reconstruction using artificial materials or xenobiomaterials. However, natural bone formation and healing using autologous cells are preferable. Therefore, development-based medicine and approaches are desired.

During the most recent decade, stem cell research has made great advances in clarifying the mechanisms of tissue development. Indeed, many stem cell researchers have focused on developmental biology and regenerative medicine, and applications of stem cells in craniofacial research have been proposed. In particular, MSCs and NCCs have been studied extensively in craniofacial research. MSCs partially originate from NCCs [7, 22–25]; therefore, some MSCs may also differentiate into neuronal cells [26–28], suggesting that specific subsets of MSCs may have the same potential as NCCs. Moreover, because MSCs are present in several adult tissues [2], they are easy to isolate and expand* in vitro*.

Notably, dental-specific MSCs have been identified in craniofacial tissues. Several research groups have reported the presence of dental MSCs in dental pulp stem cells (DPSCs) [29], stem cells from exfoliated deciduous teeth (SHED) [30], periodontal ligament stem cells (PDLSCs) [31], and stem cells from apical papilla (SCAP) [32]. These dental MSCs may have applications in degenerative and intractable diseases [33]. Furthermore, conditioned medium (CM) from dental MSCs supplies paracrine factors and may be effective for injured areas [34, 35]. Osugi et al. reported that SHED-CM promoted the growth of bone mass in a calvarial defect model. Additionally, the use of conditioned medium from dental tissue-derived MSCs is a unique approach for craniofacial regenerative medicine without cell transplantation. This approach may reduce costs and time/labor requirements and may alleviate safety concerns [36].

Bone marrow MSCs are utilized as a typical model for clinical studies and basic biology research, and they are classically defined by conventional culture, as MSCs show vigorous expansion and multipotent differentiation. However, the mechanisms of regeneration in conventional MSCs cannot be traced back to developmental fate, and it is difficult to predict which subsets of a crowded cell population contribute to the development of specific target tissues. Thus, mixed cultures of conventional MSCs may not provide consistent, predictable therapeutic outcomes from a developmental biological viewpoint. Further studies are needed to clarify the development and degeneration of craniofacial skeletal tissues using stem cells, particularly for analysis of the clonal phenotype of NCCs and MSCs.

## 3. Purified MSCs Are Partially Derived from NCCs

Cranial NCCs constitute a major part of facial tissues [3, 37]. Moreover, NCCs are localized in the neural folds of fetal tissues, migrate into various tissues, and regulate skeletal tissues [38]. Cranial NCCs form the first pharyngeal arch innerved by the trigeminal nerve and the second pharyngeal arch innerved by the facial nerve [39–41]. Then, the first and second pharyngeal arches interact with each other and form craniofacial tissues [42, 43]. Craniofacial tissues are mainly composed of bone, muscle, and tendon together with the neurovascular bundle [44]. Therefore, improving the understanding of neurogenesis and vascularization in skeletal tissues is essential for discussions of craniofacial tissue formation. To identify and track the process of cranial tissue specification, visualization of the neural crest and its derivatives is needed.

**Figure 1:**
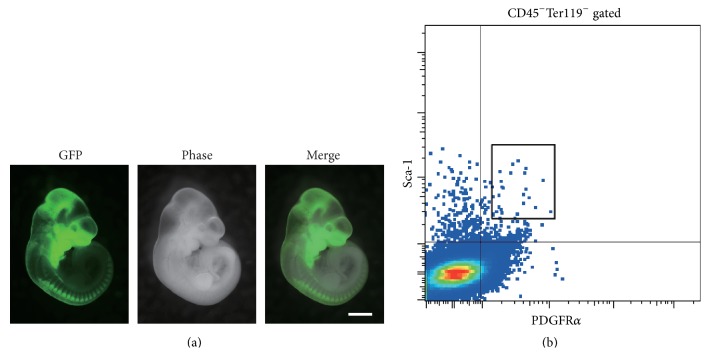
(a) Neural crest lineage labeling mouse (Wnt1-Cre/GFP) clearly demonstrates green fluorescence-positive NCCs in craniofacial tissues at embryonic day 11. Scale bar, 1 mm. (b) Murine bone marrow cells were analyzed by flow cytometry. The chart shows that cells expressing hematopoietic lineage markers (CD45 and Ter119) were negatively gated, and the highly potent murine MSC marker-expressing (P*α*S) subset is indicated by the black-colored box.

Within the last few decades, transgenic technologies have been developed that enable visualization of target cells for prospective and retrospective analyses [45]. Transgenic animal studies can be used to elucidate what and how specific cells contribute to form target tissues. This approach has also been applied to the craniofacial field, where NCCs and their derivative cells are labeled and their fates are analyzed [4, 46, 47]. This transgenic technology provides clear information on the development of NCCs in the craniofacial field and enables the visualization of NCCs and their derivatives within dental and craniofacial tissues as shown in Figure 1(a).

Some researchers have focused on cells that are easier to obtain than embryonic NCCs, such as bone marrow stem cells or pluripotent stem cells. The bone marrow is a typical source of MSCs and contains two distinct types of stem cells, hematopoietic stem cells (HSCs), and MSCs [48]. Transgenic animal studies have demonstrated that MSCs partially originate from NCCs. NCCs delaminate from neuroepithelial cells, which can differentiate into cells expressing the MSC marker platelet-derived growth factor receptor alpha (PDGFR*α*) (CD140a) [22]. Thus, neuroepithelial cells are a source of MSC differentiation. Moreover, Nagoshi et al. showed that NCCs migrate though the aorta-gonad-mesonephros region and circulate into the bone marrow, demonstrating how neural crest-derived stem cells can travel to the bone marrow [23]. Additionally, Morikawa et al. reported a subset of highly potent murine MSCs, characterized by the cell surface marker combination of PDGFR*α*, stem cell antigen-1 (Sca-1/lymphocyte activation protein; Ly-6), CD45, and Ter119 (Ly-76). This specific subset, PDGFR*α* ^+^Sca-1^+^CD45^−^Ter119^−^ (P*α*S), could be found in the bone marrow as shown in Figure 1(b) [49]. The P*α*S subset partially originates from NCCs as shown by developmental fate analysis using transgenic mice [7]. Thus, application of cell tracking systems in mice has clarified the relationship between MSCs and NCCs, supporting the application of MSCs in craniofacial skeletal tissue research.

The P*α*S subset of MSCs has been identified as the perivascular niche and has differentiation potential to both mesenchymal and neural crest lineages [7]. Indeed, the P*α*S subset promotes neural crest-derived periodontal tissue regeneration [50]. P*α*S cells are closely related to SSCs, which are identified by rigorous assays. Bone marrow MSCs also contain stem cells that can only differentiate into skeletal tissues. Thus, SSCs represent a reservoir for bone-forming cells and have the potential to shape and regulate the local microvascular network in the bone marrow [51].

Worthley et al. reported that Gremlin-1 functions as a specific marker of skeletal stem cells in the long bone marrow. Gremlin-1 is a bone morphogenic peptide (BMP) antagonist, and the transforming growth factor (TGF)-*β*/BMP signaling pathway regulates osteoblastogenesis and bone formation [52]. In Gremlin-1-overexpressing transgenic mice, Gremlin-1^+^ cells differentiate into bone, cartilage, and reticular stromal cells. Postnatally, Gremlin-1^+^ cells also contribute to development. Gremlin-1^+^ cells are not further enriched for P*α*S cells. However, the P*α*S subset also contributes to the formation of skeletal tissues [53]. Therefore, some MSCs in the P*α*S subset may have the differentiation potential of SSCs. Consistent with this notion, Gremlin regulates developing limbs, and Gremlin-1^+^ and P*α*S cell subsets have been identified in the long bone [17, 49, 54, 55]. Additionally, recent reports have shown that cranial MSCs are different from long bone marrow MSCs [56].

Zhao et al. reported that glioma-associated oncogene homolog-1 (Gli-1) is a marker of craniofacial-specific MSCs in cranial bones [57]. Gli-1^+^ cells are not coexpressed with classical MSC markers* in vivo*. Although the specific characteristics of these cells have not been defined* in vivo*, Gli-1^+^ cells show typical phenotypes of MSCs, such as vigorous expansion, expression of classical MSC markers, and differentiation to mesenchymal lineages* in vitro* [44, 57]. However, Gli-1 is not always expressed in MSCs during development, in contrast to PDGFR*α*. Notably, no direct gene regulation mechanism has been identified between Gli-1 and PDGFR*α* [58]. Gli-1 is induced by sonic hedgehog (Shh) signaling and is associated with transient Sox2 expression during tooth formation [59]. Shh signaling has also been detected in Hertwig's epithelial root sheath (HERS) and found to lead to tooth root formation [60], suggesting that Shh signaling may promote Gli-1 expression in craniofacial-specific mesenchyme. Although Gli-1^+^ cells are not present in the perivascular niche [57], MSCs in long bones are regulated by the perivascular niche [61–65]. The leptin receptor (LepR) (CD295) has been reported as an excellent marker for MSCs. LepR^+^ cells are identified around sinusoids [61, 66]. In contrast, MSCs in the cranial bone suture are found around the midline of the suture structure. This specificity of craniofacial MSCs does not correspond to MSCs in long bone marrow. These differences in murine craniofacial and long bone MSCs should be examined in greater detail in future studies.

## 4. Purified MSCs Can Be Derived from Induced Pluripotent Stem Cells (iPSCs)

To achieve the transition of stem cell research from the bench to the bedside, more studies investigating human cells are required. Human MSCs can be found in several types of tissues. The classical definition of MSCs is the same in mice and in humans, and conventional MSCs can be contaminated by other cell lineages. To avoid such problems, more studies of cell markers and selection of target cells are needed. Previously, Mabuchi et al. identified the highly specific human MSC markers, low-affinity nerve growth factor receptor (LNGFR), and thymocyte antigen 1 (THY-1) [67]. Cells expressing LNGFR and THY-1 have been identified in the bone marrow, and the combination of LNGFR and THY-1 cell surface markers characterizes a distinct subset of MSCs with a hematopoietic lineage. LNGFR^+^THY-1^+^ cells have high colony forming potential and differentiate into mesenchymal lineages. These specific cells have also been identified in decidua, fat tissues, synovium, and dental pulp [67–69]. The LNGFR^+^THY-1^+^ subset shows highly potent self-renewal and differentiation capacity both* in vitro* and* in vivo* and is also associated with the expression of other classical MSC markers, such as CD29 (integrin beta 1), CD44 (homing cell adhesion molecule; HCAM), CD73 (ecto-5′-nucleotidase), CD105 (endoglin), CD146 (melanoma cell adhesion molecule; MCAM), and CD166 (activated leukocyte cell adhesion molecule; ALCAM) [67, 69]. Efficient procedures for purification will improve the ability to analyze MSCs.

In craniofacial diseases, there is a great need for reconstruction of large areas affected by disease or injury. Thus, it is necessary to obtain large numbers of MSCs for such clinical applications. This must be achieved without damaging original tissues; therefore, specific cells derived from pluripotent stem cells based on developmental research may help to overcome this problem.

Human iPSCs were first generated in 2007 and have been used extensively in biomedical studies [70]. Human iPSCs have the capacity for self-renewal and can be expanded relatively easily. The strong potential of iPSCs can also be applied in craniofacial research. MSCs can be derived both directly from iPSCs and indirectly from neural crest like cells using specific markers and culture conditions [24, 71]. MSCs induced from iPSCs have been used in the regeneration of periodontal tissues [72]. Thus, these findings suggest that iPSC-derived MSCs have the capacity for use in craniofacial tissue regeneration.

## 5. Application of Human MSCs in Craniofacial Research

Craniofacial connective tissues originate from neural crest-derived ectomesenchyme, which is a source of many craniofacial bone and cartilage structures. Umeda et al. reported the generation of ectomesenchymal cells through neural crest-like progeny from human iPSCs [73]. Sensory innervation is necessary for maintaining sound bone. In dentistry, sensory innervation of craniofacial tissues involves the trigeminal nerve. Previously, several methods were reported for induction of peripheral nerve formation using NCCs [74, 75]. Application of sensory neuronal cells to craniofacial skeletal tissues requires neural crest-derived craniofacial-specific sensory neuronal induction. Dincer et al. reported that the craniofacial placode can be used to identify the craniofacial trigeminal nerve [76]. However, induction procedures for craniofacial target tissues are a relatively new approach in regenerative medicine and disease-specific iPSC technology. Further studies are needed to provide clear information on neural crest biology and craniofacial specificity. Methods for induction of target cells must take advantage of the generation of a sufficient number of cells, and induction based on the basic knowledge of developmental biology may provide an evidence-based approach for application of stem cells in the clinical field.

For application of human iPSC-derived cells in the regeneration of craniofacial tissues, prevention of teratoma formation represents a major challenge. iPSC lines show variations in the patterns of teratoma formation in iPSC-derived neural progenitor cells [77, 78]. Surprisingly, despite the exclusion of pluripotent markers, iPSCs may form teratomas in some cases. Lee et al. reported that iPSC-derived neural crest-derived stem cells, which exhibit downregulation of polysialic acid-neural cell adhesion molecule (PSA-NCAM), tend to form teratomas [79]. These findings suggest the importance of basic and preclinical studies of iPSC-derived NCCs. Recent studies have shown the safety of iPSC-derived cells in preclinical models [80]. Reconstruction of the target craniofacial tissues in nonhuman primate models is essential before human clinical studies using iPSCs can be initiated. Prescott et al. showed that* cis*-regulatory divergence is associated with differences in quantitative expression in human and chimpanzee cranial NCCs derived from iPSCs [81]. It is quite important that this study demonstrated the novel application of iPSC-derived NCCs for analyzing evolutionary cellular anthropology in the context of craniofacial development.

Thus, iPSC technology may facilitate future applications in regenerative medicine if the risk of iPSC-derived tumor formation can be minimized.

## 6. Conclusion 

Skeletal tissues are composed of bone, cartilage, and tendon. These mesenchymal tissues are generated from NCC-derived MSCs and exhibit neurogenesis and neovascularization. MSCs are part of the perivascular niche and overlap with neural crest-derived stem cells [25]. MSCs have potential for differentiating into skeletal cells, neuronal cells, and endothelial cells, suggesting that MSCs may be useful for craniofacial tissue regeneration. Indeed, MSCs have been applied for the treatment of craniofacial diseases, such as periodontitis and osteonecrosis of the jaw in small animal models [82, 83]. The potential of stem cells is typically demonstrated using mice and rats because they are easy to breed and handle, and there is a variety of well-established disease models recognized by the scientific community. However, the craniofacial anatomy of these animals is different from that of humans. Several research groups reported stem cell approaches using larger animals such as pigs, dogs, and chimpanzees [32, 81, 84]. In animal studies, establishment of live cell imaging systems can be used to clearly visualize the potential for migration and differentiation. Such imaging systems have been utilized in the craniofacial field [69, 85]. Demonstration of cell tracking in large animal models such as nonhuman primates provides essential evidence for human preclinical studies that expected to take place in the near future.

Human clinical application of stem cells has already started [86, 87]; however, the current protocols for clinical application mainly utilize conventionally cultured cells. Conventional MSCs contain various types of cells within adherent culture, resulting in contamination of the MSCs with other cell lineages. Moreover, MSCs show great differences in characteristics between long bones and craniofacial tissue, and these differences should be evaluated in detail in future studies.

For further analyses of the applications of MSCs in humans, the developmental fate of human MSCs must be elucidated. Sufficient quantities of purified MSCs from adult tissues for reconstruction of large spaces in the craniofacial region are difficult to collect. Human iPSC technology may be used to overcome this problem. Furthermore, more analyses of MSCs, NCCs, SSCs, and iPSCs are required based on a developmental biological approach, which will be expected to provide evidence-based methods for the treatment of various craniofacial diseases.
